# An Unusual Perianal Presentation of a Tailgut Cyst

**DOI:** 10.7759/cureus.24953

**Published:** 2022-05-12

**Authors:** Ramya Suresh, Vijay Shankar S.

**Affiliations:** 1 Department of Pathology, Adichunchanagiri Institute of Medical Sciences, Mandya, IND

**Keywords:** retrorectal cystic hamartoma, rare, malignant transformation, congenital, embryological remnant, perianal, tailgut cyst

## Abstract

Tailgut cysts or retrorectal cystic hamartomas are rare, congenital, development lesions arising from the remnants of the hindgut during embryogenesis. It is most often misdiagnosed due to its rarity, variable clinical presentation, and malignant potential. The following report describes an unusual case of a tailgut cyst in a 60-year-old male with a history of a perianal mass for 12 years. Surgical resection was done, and histopathological examination revealed a multiloculated cyst filled with brownish fluid, grossly, and a cyst lined by various epithelia such as stratified squamous epithelium, pseudostratified columnar epithelium, and flattened to cuboidal and mucin-secreting columnar epithelium along with cyst wall made up of bundles of smooth muscles, microscopically. Several lesions mimic a tailgut cyst and need to be excluded from the differentials. Although no malignancy was documented in this case, these cysts have been known to undergo malignant transformation into adenocarcinoma and neuroendocrine carcinoma, to name a few. This warrants a thorough and accurate histopathological assessment and mandatory follow-up.

## Introduction

Tailgut cysts or retrorectal cystic hamartomas are rare, congenital, hamartomatous lesions that arise from the persistent embryological remnants of the postanal gut (hindgut) that fail to regress completely [1]. They occur in all age groups, more commonly in middle-aged women and mostly in the retrorectal (presacral) space. This space is defined anteriorly by the rectum, posteriorly by the sacrum, superiorly by the peritoneal reflection, inferiorly by the levator ani and coccygeus muscles, and laterally by the ureter and iliac vessels [2]. They rarely appear in anterior rectal, perianal, perirenal, and posterior sacral regions [3].

Tailgut cysts are benign and need to be distinguished from other congenital malformations and benign and malignant neoplasms that occur in this location [4]. Owing to the rarity of this condition and its nonspecific presentation, the diagnosis is frequently late or misdiagnosed, leading to malignant transformation of the lesion, the majority being adenocarcinomas, neuroendocrine carcinomas, and carcinoid tumors [2,3,5,6]. In this report, we document a case of tailgut cyst arising in the perianal region.

This case was previously presented as a poster at KAPCON 2021 Virtual Conference.

## Case presentation

A 60-year-old male patient presented with a painless, perianal lesion of 12 years in duration. No history of rectal bleeding/discomfort, constipation, urinary problems, or fever was reported. The physical examination revealed a polypoidal, skin-covered mass in the perianal region that was soft to touch, non-tender, and not adhered to surrounding structures. The patient underwent resection of the tumor with no significant intraoperative findings or intra-/postoperative complications. The specimen was sent for histopathological examination.

**Figure 1:**
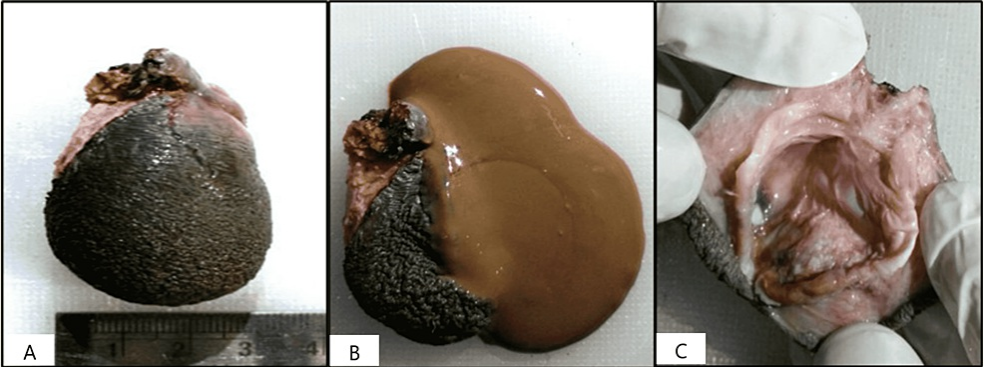
Gross features of the received specimen of a tailgut cyst. A: A polypoidal, skin-covered, soft/cystic gross specimen of a tailgut cyst measuring 5 × 3 × 1 cm. B: Cut surface: brownish fluid expelled from the tailgut cyst. C: Cut surface: multiloculated cyst with trabeculations.

Grossly, the resected skin-covered specimen measuring 5 × 3 × 1 cm showed a multiloculated cyst with trabeculations and expelled brownish material (Figure 1A-1C).

**Figure 2:**
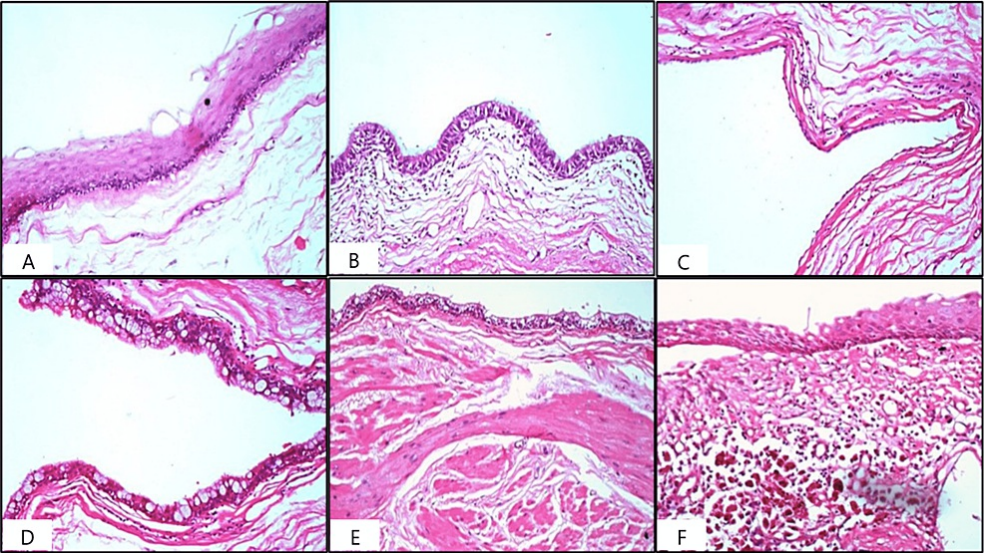
Histopathological examination revealed a cyst lined by various epithelia. A: Stratified squamous epithelium (H&E, 40×). B: Pseudostratified columnar epithelium (H&E, 40×). C: Flattened to cuboidal epithelium (H&E, 40×). D: Mucin-secreting epithelium (H&E, 40×). E: Cyst wall containing scattered, discontinuous smooth muscle bundles (H&E, 40×). F: Pigment-laden macrophages and chronic inflammatory cell infiltrates (H&E, 40×). H&E: hematoxylin and eosin

Microscopically, the cyst was lined by various epithelia, including stratified squamous epithelium, pseudostratified columnar epithelium, and flattened to cuboidal and mucin-secreting columnar epithelium. The cyst wall contained bundles of smooth muscles. Areas of pigment-laden macrophages and chronic inflammatory cell infiltrates were noted. Skin adnexal structures, neuronal elements, bone, and cartilage were not identified in the cystic areas. No atypical/malignant cells were identified (Figure 2A-2F).

Based on these findings, the case was diagnosed as a tailgut cyst.

## Discussion

The origin of the tailgut cyst is hypothesized to arise from the remnants of the normally regressing hindgut tail around the eighth week of embryogenesis [2,5].

Tailgut cysts have various nonspecific clinical manifestations, making diagnosis difficult [7,8]. Most of the patients are asymptomatic or present with nonspecific mass effect symptoms such as abdominal pain, defecation difficulty, constipation, or even an abdominal mass [9,10]. Occasionally, it can obstruct the pelvic outlet, leading to dystocia or sciatica. Rarely, patients also complain of vaginal pain.

They may get secondarily infected and misdiagnosed as a pilonidal cyst, a perianal/anorectal fistula, or a recurrent retrorectal abscess [6]. Signs of an underlying infection are indistinct cyst margins with nodular wall thickening, intra-cystic vegetations, and surrounding lymphadenopathy [5].

The differential diagnoses of a tailgut cyst include anal gland cyst, duplication cyst, epidermal cyst, sacral meningocele, and teratoma/dermoid cyst [6]. The location in the anal mucosa is suggestive of an anal gland cyst [5]. Duplication cysts are unilocular, commonly lined by gastric, colonic, or respiratory epithelium containing villi, crypts, and glands with a well-formed two-layer smooth muscle wall [3,4]. Epidermoid cysts have squamous lining epithelia [3]. A teratoma or dermoid cyst is unilocular, lined by stratified squamous epithelium along with adnexal structures, and the wall has no smooth muscles [4].

Hjermstad and Helwig, the first authors to report a tailgut cyst, specified two histological criteria for the diagnosis of a tailgut cyst: (a) the epithelial lining of the cysts must contain transitional and/or glandular type (columnar) of epithelium, with or without a stratified squamous component, and (b) the underlying stroma must compose fibrous tissue with scattered and discontinuous smooth muscle bundle fibers, i.e., it must lack a well-defined muscular layer [10]. Both the criteria were fulfilled in our case.

A large majority of tailgut cysts are benign; however, malignant transformation, although rare, has been reported in several cases. They include adenocarcinomas, carcinoid tumors, neuroendocrine carcinomas, endometrioid carcinomas, adenosquamous carcinomas, squamous cell carcinomas, and sarcomas, in decreasing order of frequency [1-6]. Evidence suggests that the dysplasia-carcinoma sequence, initially established in the colon, can also exist in tailgut cysts [6].

Given the limitations of imaging techniques to make a definitive diagnosis, a preoperative biopsy could facilitate optimal surgical planning [11]. However, it is not recommended as there are chances that it may contain only fibrous tissue without epithelia or only one epithelium, and the malignant foci may not be sampled. Additionally, there is a risk of infection, and malignant cells can spill into the abdominal cavity and lead to tumor spread [1,2,5].

Complete surgical excision followed by histopathological examination is a must to eliminate the risk of recurrence, hemorrhage, infection, compression, and malignant transformation [6,12]. Postsurgical adjuvant radiation therapy with or without chemotherapy is suggested in cases of malignancy to kill residual tumor cells, prevent metastasis, and improve patient prognosis [2,13].

## Conclusions

Tailgut cysts are rare, congenital lesions derived from vestigial elements of the embryonic hindgut. The clinical diagnosis is difficult because it is mostly asymptomatic or manifests with nonspecific mass effect symptoms or neurological symptoms. They are usually misdiagnosed and go into complications including malignant transformation. Complete surgical resection is mandatory upon diagnosis, followed by histopathological examination, and subsequent follow-up must be ensured.
